# Caveolin-1 defines astrocyte mechanobiology in the glial lamina of the optic nerve head

**DOI:** 10.3389/fncel.2026.1909639

**Published:** 2026-10-07

**Authors:** Anja K. Hoffmann, Anna Dlugosch, Michelle C. Geigenfeind, Amelie Seemann, Michael H. Elliott, Rudolf Fuchshofer, Andrea E. Dillinger

**Affiliations:** 1 Institute of Human Anatomy and Embryology, University of Regensburg, Regensburg, Germany; 2 Institute of Neuroanatomy, University Medical Center Hamburg-Eppendorf, Hamburg, Germany; 3 Department of Biochemistry & Physiology, The University of Oklahoma Health Campus, Oklahoma, OK, United States; 4 Dean A. McGee Department of Ophthalmology, The University of Oklahoma Health Campus, Oklahoma, OK, United States

**Keywords:** extracellular matrix, glaucoma, IOP, mechanical load, optic nerve

## Abstract

The optic nerve head is the principal site of retinal ganglion cell axonal injury in primary open angle glaucoma, where astrocyte mechanobiology and extracellular matrix remodeling are thought to play critical roles in disease progression. Caveolae, which are mechanosensitive plasma membrane invaginations formed by Caveolin and Cavin proteins, are key regulators of cellular responses to mechanical stress, yet their involvement in optic nerve head pathophysiology has not been defined. Here, we characterize the presence, regulation, and mechanobiological relevance of Caveolin and Cavin proteins in murine optic nerve astrocytes *in vitro* and *in vivo*. Primary optic nerve astrocytes exhibited classical caveolar structures and expressed Caveolin-1, Caveolin-2 and multiple Cavins. *In vivo*, Caveolin-1 is highly enriched in astrocytes of the glial lamina. Glaucoma-associated factors CCN2/CTGF and TGF-β2 significantly increased Caveolin-1 and Cavin-1 expression in optic nerve astrocytes, while CCN2/CTGF knockdown reduced Caveolin-1 expression. Increasing substratum stiffness enhanced Caveolin-1 expression and altered the subcellular localization of Caveolin-1. Furthermore, Caveolin-1 mRNA expression was increased in the optic nerve head of a glaucoma mouse model, while its protein levels were elevated in the entire optic nerve of the transgenic mice. Together, these findings identify caveolae and Caveolin-1 as mechanosensitive components of optic nerve astrocytes and suggest their involvement in biomechanical and molecular alterations of the glaucomatous optic nerve head. This work provides the first evidence linking astrocyte mechanobiology in the glial lamina to Caveolin-1, highlighting a potential novel contributor to glaucoma pathogenesis.

## 1 Introduction

The optic nerve head represents the principal site where retinal ganglion cell axons exit the eye, and its architecture differs markedly between species (Anderson, 1969; Howell et al., 2007; May and Lutjen-Drecoll, 2002; Quigley, 1987; Quigley et al., 1983; Radius and Bade, 1982; Sun et al., 2009). In humans, this region contains the lamina cribrosa, a mesh-like arrangement of connective tissue and elastic fibers lined by a dense network of astrocytes and lamina cribrosa cells. In contrast, mice possess a glial lamina composed almost exclusively of astrocytes that lacks the connective tissue scaffolding found in primates (Anderson, 1969; Howell et al., 2007; May and Lutjen-Drecoll, 2002; Quigley et al., 1983; Sun et al., 2009). The laminae in general helps distribute and resist the biomechanical forces by the intraocular pressure (IOP), protecting the nerve fibers. Astrocytes of both structures provide essential support to neurons through multiple mechanisms, including signaling across cellular networks and regulation of neurovascular coupling (Boal et al., 2021; Fields et al., 2015; Petzold and Murthy, 2011; Stackhouse and Mishra, 2021; Verisokin et al., 2021). In primary open-angle glaucoma (POAG), axonal injury occurs at this site and leads to progressive retinal ganglion cell loss (Quigley, 2011). Elevated IOP is a major risk factor for this degeneration, and numerous clinical trials have demonstrated that lowering IOP slows disease progression (Collaborative Normal-Tension Glaucoma Study Group, 1998; The AGIS Investigators, 2000; Danias and Podos, 1999; Gordon et al., 2002; Leske et al., 2003, 1999). Throughout the optic nerve head, particularly within the lamina cribrosa or glial lamina, astrocytes exhibit pronounced reactivity during POAG (Anderson, 1969; Howell et al., 2007; May and Lutjen-Drecoll, 2002; Quigley, 1987; Quigley et al., 1983). Whether these changes are ultimately protective or detrimental for axonal health remains uncertain. As the disease advances, the organization of the laminar architecture becomes progressively altered, including shifts in the quality and quantity of the extracellular matrix components mainly induced by growth factors like transforming growths factor (TGF)-β2 (Zode et al., 2011). These changes modify the biomechanical properties of the tissue and thereby influence the mechanobiological environment to which axons and glial cells are exposed (Gottanka et al., 2005; Hopkins et al., 2020; Pena et al., 2001, 1998; Tektas et al., 2010). Besides other structures, like mechanosensitive ion canals, caveolae are known to be responsible for mechanosensation and -transduction (Del Pozo et al., 2021). The main components of caveolae are members of two protein families: caveolins and cavins. Caveolins are small integral membrane proteins with the N- and C-termini facing the cytoplasm, so they get a hairpin conformation. There are three members of this family: Caveolin-1, Caveolin-2 and Caveolin-3. Caveolin-1 and Caveolin-2 are expressed in most tissue, while Caveolin-3 is exclusively expressed in muscle tissue (Del Pozo et al., 2021). Caveolin-1 is essential for caveolae formation, as its depletion results in the complete absence of caveolae (Drab et al., 2001; Razani et al., 2002). Cavins are peripheral membrane proteins that form oligomeric complexes with Caveolin-1 in parts of the plasma membrane to form caveolae (Hansen and Nichols, 2010; Parton et al., 2020; Stoeber et al., 2016). In glaucoma, the extracellular matrix changes in quality and quantity, and thereby influences mechanical cues sensed by resident cells (Gottanka et al., 2005; Hopkins et al., 2020; Pena et al., 2001, 1998; Tektas et al., 2010). In the cells mechanical cues can activate signal transduction pathways and an increase in mechanoprotection capacity, both transferred by caveolae (Del Pozo et al., 2021). Until now it is not known if caveolae and these processes are involved in optic nerve pathogenesis in glaucoma, but genome-wide association study for POAG identified significant associations with single nucleotide polymorphisms (SNPs) between the CAV1 and CAV2 genes on chromosome 7q31 (Thorleifsson et al., 2010; Wiggs et al., 2011). To this end, we investigate the presence of caveolae and their proteins in primary murine optic nerve astrocytes, the resident cells of the optic nerve *in vitro* and in the mouse *in vivo*. Furthermore, we studied how glaucoma-relevant factors, like CCN2/CTGF and TGF-β2 affect the expression of Caveolin-1 and Cavin-1. Finally, we analyzed how Caveolin-1 is regulated by substratum stiffness in murine optic nerve astrocytes *in vitro* and in the optic nerve of a glaucoma mouse model.

## 2 Results

### 2.1 Expression and Localization of Caveolins and Cavins in Optic nerve astrocytes

Using scanning electron microscopy primary murine optic nerve astrocytes showed caveolae structure, which are comparable in appearance and size to caveolae found in a brain endothelia cell line (bEnd5), which were used as a positive control for caveolar structures (Figure 1A). In endothelial cells, caveolae represent small, surface-associated membrane indentations characterized by a distinctive bulb-and-neck morphology and a typical diameter in the range of 50–100 nm, with most structures measuring approximately 60–80 nm. The caveolae structure we found in primary murine optic nerve astrocytes showed a similar size (Frank et al., 2003; Wang et al., 2009). However, to investigate whether cultured primary optic nerve astrocytes also express members of the Caveolin and Cavin protein families we performed real-time RT-PCR (Figure 1B). We could identify the expression of Caveolin-1, Caveolin-2, Cavin-1, Cavin-2, Cavin-3 and Cavin-4 (Figure 1B). Cavoelin-3, known to be predominantly expressed in muscle cells, was not detected in primary murine optic nerve astrocytes (Figure 1B). We then analyzed the most abundant and essential caveolae formation family members, Caveolin-1 and Cavin-1, in more detail. Immunocytochemical staining could verify the presence of both proteins in primary murine optic nerve astrocytes *in vitro* (Figure 1C). These results showed that the proteins Caveolin-1 and Cavin-1 are highly present and that caveolae are formed in murine optic nerve astrocytes.

**Figure 1:**
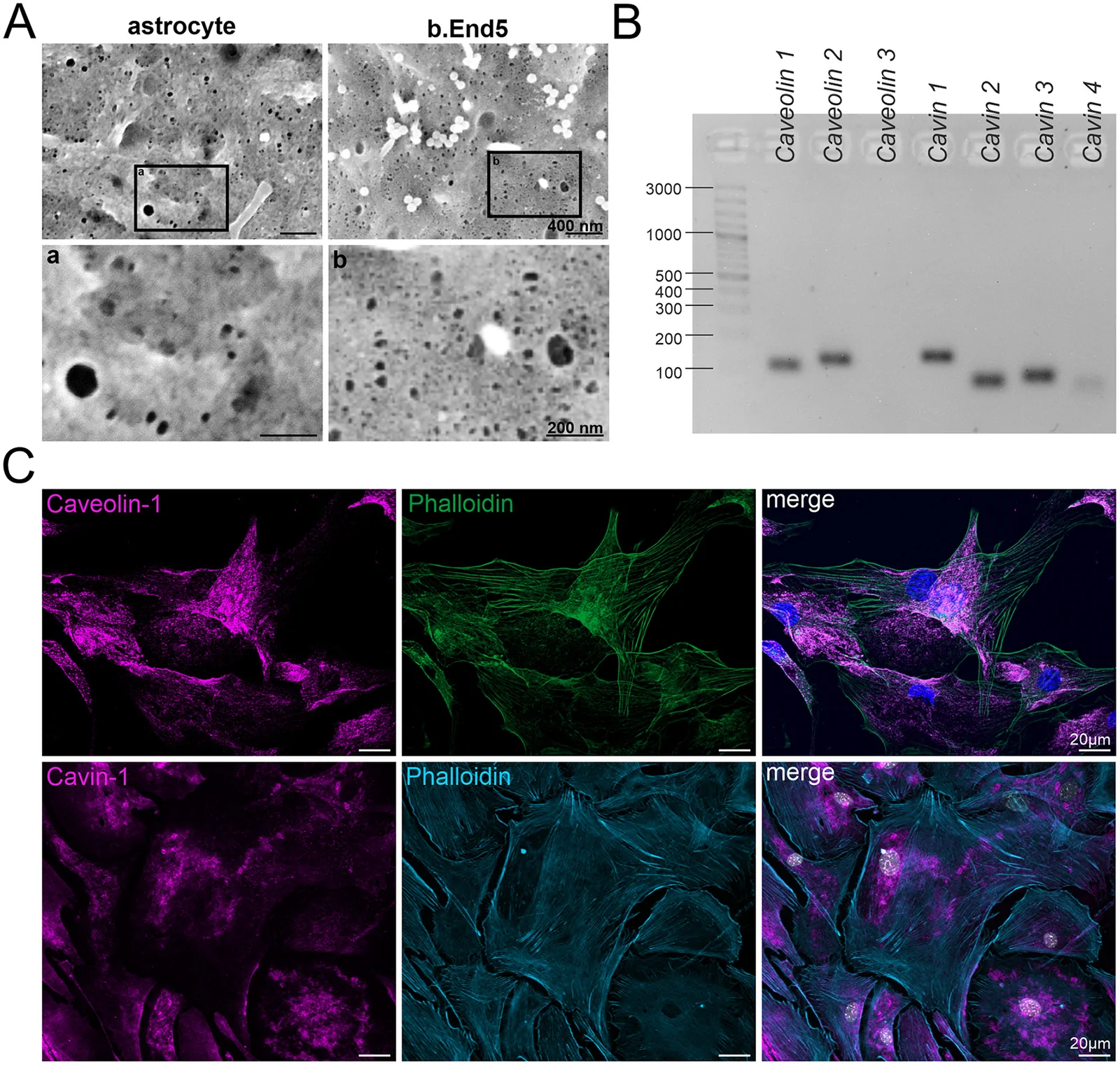
Caveolae forming Caveolins and Cavins are abundantly expressed in primary murine optic nerve astrocytes *in vitro*. **(A)** Scanning electron microscopy images showing caveolae on the surface of a murine optic nerve astrocyte and a b.End5 endothelial cell. Higher magnification images **(a–b)** reveal comparable caveolar morphology in both cell types. Scale bars: 400 nm, 20 nm; **(B)** Agarose gel electrophoresis of amplified real-time RT-PCR products for *Caveolin 1, Caveolin 2, Caveolin 3, Cavin 1, Cavin 2, Cavin 3*, and *Cavin 4* in murine optic nerve astrocytes **(C)** Immunofluorescent labeling of Caveolin-1 (upper row, magenta) and Cavin-1 (lower row, magenta) with phalloidin co-labeling (green/cyan) in murine optic nerve astrocytes *in vitro*. Cell nuclei: Dapi (blue), Scale bars: 20 μm.

Analysis of Caveolin-1 expression in wildtype mice at the age of 8 weeks showed a highly concentrated localization in the region of the glial lamina. We found Caveolin-1 predominantly localized in the glial lamina, to a lower extent in the prelaminar region of the optic nerve and it was almost completely absent in the post-laminar part of the optic nerve (Figure 2). Immunohistochemical staining in combination with GFAP, a specific marker for astrocytes in the glial lamina identified Caveolin-1 strongly localized to astrocytes (Figure 2). Moreover, we could detect intense Caveolin-1 staining in the vasculature of the optic nerve (Figure 2). Interestingly, the weak Caveolin-1 signal in the post-laminar region did not localize to astrocytes and therefore is more likely attributed to small blood vessels within the optic nerve (Figure 2).

**Figure 2:**
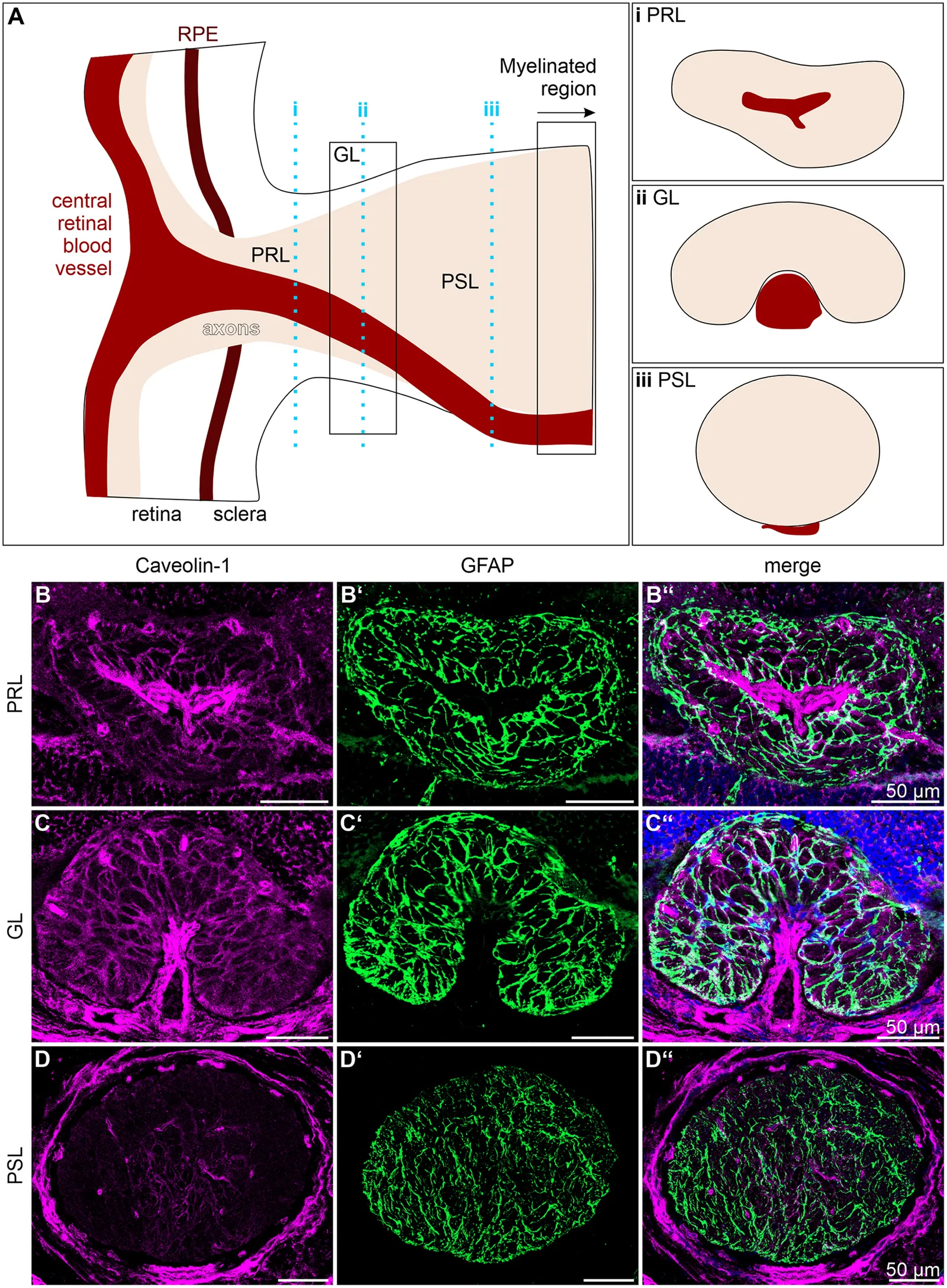
Astrocytes of the murine glial lamina express Caveolin-1 *in vivo*. **(A)** Schematic illustration of a meridional section through the optic nerve head of the mouse eye, depicting the prelaminar region (PRL), glial lamina (GL), and postlaminar region (PSL). Panels (**i-iii**) depict the expected morphology of the optic nerve tissue in tangential sections through PRL (**i**), GL (**ii**), and PSL (**iii**). **(B-D)** Tangential sections of the murine optic nerve under immunofluorescence labeling for Caveolin-1 (magenta) and GFAP (green): **(B)** PRL **(C)** GL, and **(D)** PSL. Cell nuclei: Dapi (blue). Scale bars: 50 μm.

### 2.2 CCN2/CTGF and TGF-β2 modulate Caveolin-1 expression and protein level

As it is known that CCN2/CTGF and TGF-β2 induce expression and protein levels of various extracellular matrix molecules related to a glaucomatous phenotype and astrocyte reactivity, we investigated their effect on members of the Caveolin and Cavin protein family.

We analyzed the expression of *Caveolin-1* and *Cavin-1* after the treatment with CCN2/CTGF and TGF-β2 for 24 h (Figure 3) in primary murine optic nerve astrocytes. We could show that CCN2/CTGF (50 ng/ml) and 1 ng/ml TGF-β2 significantly increased Caveolin-1 mRNA expression compared to vehicle treated control cells (Figure 3A). Western blot analysis also showed that protein levels of Caveolin-1 were significantly increased after treatment with CCN2/CTGF (50 ng/ml and 100 ng/ml) and TGF-β2 for 24 h compared to untreated control cells (Figure 3B). Similar results were obtained for Cavin-1. However, the mRNA expression of *Cavin-1* was not significantly altered after treatment with CCN2/CTGF or TGF-β2 for 24 h compared to untreated control cells (Figure 3C). Western blot analysis showed that Cavin-1 protein levels were significantly increased after treatment with 50 ng/ml or 100 ng/ml CCN2/CTGF, or 1ng/ml TGF-β2 compared to control murine optic nerve astrocytes (Figure 3D). The treatment of CCN2/CTGF and TGF-β2 had no effect on the expression of *Cavin-2* and *Cavin-3* (data not shown). To analyze the relationship of CCN2/CTGF and the Caveolin and Cavin protein family, we performed siRNA interference to downregulate CCN2/CTGF (Figure 3E). We could significantly reduce the expression of *Ccn2/Ctgf* by *Ccn2/Ctgf* specific siRNA compared to cells transfected with a scrambled control siRNA (Figure 3E). Interestingly, we could show that the reduced expression of *Ccn2/Ctgf* in murine optic nerve astrocytes led to a significantly decreased expression of *Caveolin-1* (Figure 3F). Interestingly, the expression of *Cavin-1* was unaffected by the reduced *Ccn2/Ctgf* expression (Figure 3F). Adenoviral transduction of Cre recombinase in murine optic nerve astrocytes derived from *Ctgf*^Coin/Coin^ mice resulted in a significant down regulation of *Ctgf/Ccn2*, compared to adenoviral transduction of the control construct (Ad5-GFP; Supplemental Figure 1A). Finally, when analyzing the protein levels of the two factors, we found that reduced expression of *Ccn2/Ctgf* increased the protein levels of Caveolin-1 (Figure 3G) and decreased the levels of Cavin-1 (Figure 3H).

**Figure 3:**
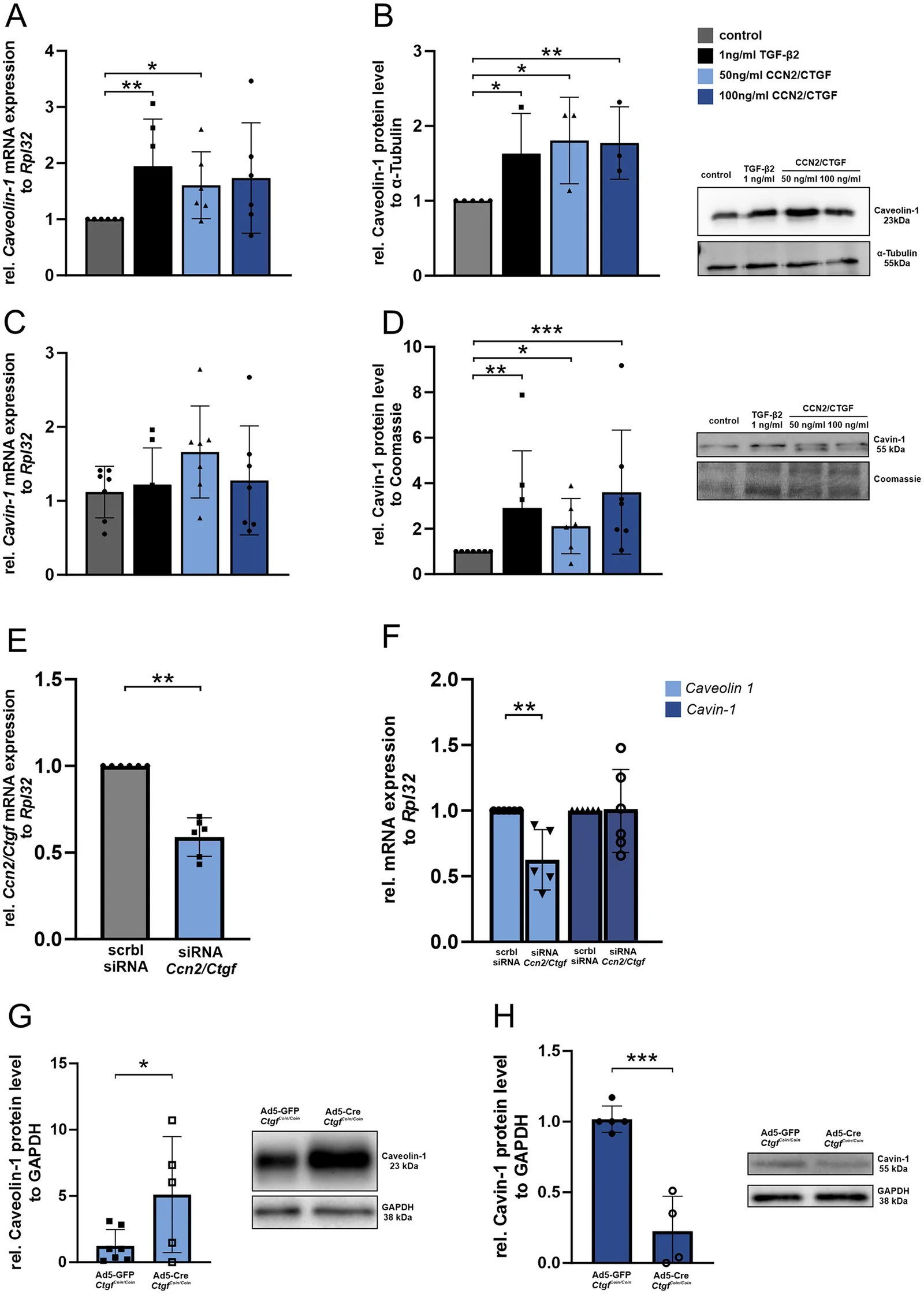
CCN2/CTGF and TGF-β2 modulate the expression of Caveolin-1 and Cavin-1 in murine optic nerve astrocytes *in vitro*. **(A-D)** Quantification of Caveolin-1 **(A, B)** and Cavin-1 **(C, D)** mRNA and protein levels in murine optic nerve astrocytes following treatment with 1 ng/ml TGF-β2 or, 50 or 100 ng/ml CCN2/CTGF, assessed by real-time RT-PCR and Western blot analysis. Kruskal-Wallis test followed by Dunn‘s multiple comparison was used for statistical analysis. **(E)** *Ccn2/Ctgf* mRNA levels following siRNA-mediated silencing compared with a scrambled (scrbl) control. Mann-Whitney test was used for statistical analysis. **(F)** *Caveolin-1* and *Cavin-1* mRNA levels determined by real-time RT-PCR following siRNA-mediated silencing of CCN2/CTGF compared with the scrbl control group. Mann-Whitney test was used for statistical analysis. **(G)** Caveolin-1 protein levels in murine optic nerve astrocytes of *Ctgf*^*Coin*/*Coin*^ mice following adenoviral *Ccn2/Ctgf* reduction determined by western blot analysis. Unpaired t-test was used for statistical analysis. **(H)** Cavin-1 protein levels in murine optic nerve astrocytes of *Ctgf*^*Coin*/*Coin*^ mice following adenoviral *Ccn2/Ctgf* reduction assessed by western blot analysis. Unpaired *t*-test was used for statistical analysis. GAPDH, α-Tubulin and Coomassie staining were used for protein normalization, and *Rpl32* was used as the housekeeping gene. Data presented as mean ± SD. *P ≤ 0.05; ***P < 0.001.

### 2.3 Increasing substratum stiffness influences Caveolin-1 expression and localization

In glaucoma biomechanical alterations due to increased intraocular pressure and changes in the quality and quantity of the extracellular matrix have been reported in a variety of glaucoma mouse models, including the βB1-CTGF1 mouse (Dillinger et al., 2022; Junglas et al., 2012). To test whether substratum stiffness has an effect on Caveolin-1 expression in murine optic nerve astrocytes, we cultured primary murine optic nerve astrocytes on cell culture substratum with different stiffness (8-10 kPa and 50-60 kPa). Murine optic nerve astrocytes grown on cell culture plates with high substratum stiffness showed a significantly enhanced Caveolin-1 expression compared to cells cultured on softer substratum (Figure 4A). To more robustly analyze the stiffness-dependence of the changes in Caveolin-1, subsequent experiments were performed on 50kPa substratum alongside with glass substratum (70GPa). Immunocytochemical staining of murine optic nerve astrocytes cultured on substratum with different stiffness (50kPa, glass 70GPa) against Caveolin-1 showed a change in localization (Figure 4B). Vinculin immunoreactivity was used to visualize that murine optic nerve astrocytes sense the changing stiffness of their surrounding tissue, which can be detected with the increase in vinculin on focal adhesions. On softer substratum, Caveolin-1 appeared to be more evenly distributed throughout the cytoplasm and along the cell membrane. With increasing substratum stiffness, Caveolin-1 staining appeared more punctate and tended to accumulate in the vicinity of focal adhesions, showing partial overlap with vinculin staining in murine optic nerve astrocytes (Figure 4B). This observation was further supported by a significant increase in the relative % area of Caveolin-1 immunofluorescent labeling in astrocytes cultured on glass compared to those cultivated on 50 kPa substratum (Figure 4C). Furthermore, cultivation of primary optic nerve head astrocytes on substratum with increasing stiffness (8-50 kPa) and additional siRNA mediated silencing of *Caveolin-1* (Supplemental Figure 1B) demonstrated a trend toward an increase in collagen IV protein levels (Supplemental Figure 2A) as well as a trend toward an increase in the pFAK/FAK protein ratio (Supplemental Figure 2B).

**Figure 4:**
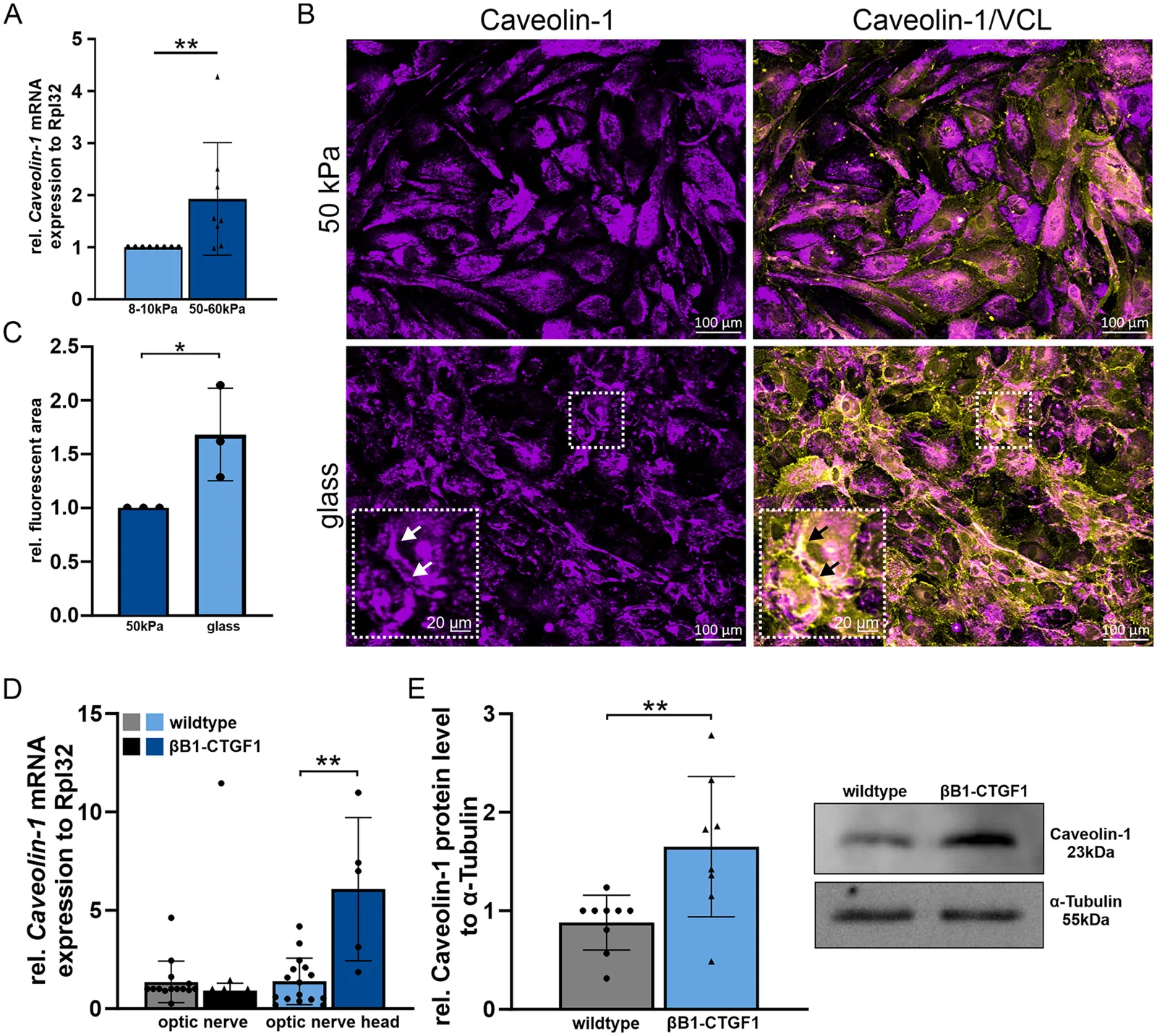
Caveolin-1 is regulated by substratum stiffness and in the glaucomatous optic nerve head **(A**) Real-time RT-PCR analysis of *Caveolin-1* mRNA levels in murine optic nerve astrocytes cultured on 8-10kPa and 50-60kPa substratum. Mann-Whitney test was used for statistical analysis. **(B)** Immunofluorescent labeling of Caveolin-1 (magenta) and vinculin (VCL, yellow) in murine optic nerve astrocytes cultured on 50kPa substratum (upper row) or glass slides (lower row). White/black arrows highlight focal adhesions in larger magnification images. Scale bars: 100 μm. **(C)** Quantification of the relative %area of immunofluorescent labeling against Caveolin-1 in murine astrocytes cultered on 50kPa substratum of glass slides. One-sample *t*-test was used for statistical analysis. **(D)** Real-time RT-PCR experiments of *Caveolin-1* mRNA levels in the optic nerve and optic nerve head of βB1-CTGF1 mice compared to wildtype littermates. *Rpl32* served as the housekeeping gene. Mann-Whitney test was used for statistical analysis. **(E)** Caveolin-1 Protein levels in optic nerve tissue of βB1-CTGF1 mice compared to wildtype littermates determined by Western Blot experiments relative to α-Tubulin. Unpaired t-test was used for statistical analysis. Data presented as mean ± SD. *P ≤ 0.05; **P < 0.01.

We could show that Caveolin-1 and Cavin-1 are expressed in murine optic nerve astrocytes. Thus, we analyzed whether there is a dysregulation of these proteins under glaucomatous conditions in the βB1-CTGF1 mice, a glaucoma mouse model (Quigley, 1987; Verisokin et al., 2021). Real-time RT-PCR analysis of optic nerve head tissue showed a dramatic significant increase in Caveolin-1 mRNA expression in the transgenic mice compared to wildtype littermates (Figure 4C). However, no changes in Caveolin-1 mRNA expression were detected in optic nerve tissue of βB1-CTGF1 mice compared to wildtype controls (Figure 4C). In contrast, Caveolin-1 protein was significantly increased in the entire optic nerve tissue, including the optic nerve head, in transgenic animals compared to their wildtype littermates (Figure 4D).

## 3 Discussion

The results of this study demonstrate that Caveolae and thereby Caveolin1, present in astrocytes of the optic nerve head, may play a considerable role in glaucomatous changes observed in the optic nerve head. This suggestion is based on the facts that (1) the caveolae proteins Caveolin-1, Caveolin-2, Cavin-1, Cavin-2, Cavin-3 and Cavin-4 are expressed in murine optic nerve astrocytes, (2) there is caveolae formation in these cells, (3) Caveolin-1 is almost exclusively present in the astrocytes of the glial lamina, (4) glaucoma-relevant factors like CCN2/CTGF and TGF-β2 enhance Caveolin-1 and Cavin-1 levels, (5) Caveolin-1 is enhanced by increasing substratum stiffness, and 6) the glaucomatous optic nerve head shows increased levels of Caveolin-1.

In POAG, the injury of retinal ganglion cell axons occurs within the lamina cribrosa in humans and in the glial lamina in mice. The mechanism by which the axon injury leads to the RGC death is not completely understood until now and studies suggest a combination of numerous complex processes, including IOP-induced biomechanical stress and strain at the lamina cribrosa, disruption of the axonal transport and ischemia (Cioffi et al., 2004; Cone-Kimball et al., 2013; Downs et al., 2005; Pease et al., 2000). However, substantial evidence supports a pivotal role for optic nerve head astrocyte mechanobiology (Ethier and Herberg, 2025). Biomechanical properties of the peripapillary sclera can be chang in relation to IOP. This is indicated by the fact that an experimentally elevated IOP leads to an increased scleral stiffness, similar to the observation in glaucomatous human eyes (Cone-Kimball et al., 2013; Downs et al., 2005; Nguyen et al., 2013). Astrocytes, forming the glial lamina in the murine optic nerve head, are in direct contact with the peripapillary sclera with their processes (Sun et al., 2009). Their capability to sense changes in stiffness of the surrounding environment points toward the evidence that optic nerve astrocytes are able to directly transfer scleral wall tension to the passing optic nerve axons (Dillinger et al., 2022; Quigley and Cone, 2013). Furthermore, they react to IOP elevation *in vivo* (Morrison et al., 2016; Tehrani et al., 2016, 2019, 2014). However, it has to be elucidated whether this is a direct effect of the elevated IOP or occurs secondary due to biomechanical alterations in the surrounding tissue. Nevertheless, this data indicates that mechanosensing and mechanotransduction are important mechanisms in optic nerve head pathologies. Interestingly, the expression of members of the mechanosensitive transient receptor potential (TRP) channels family, and Piezo1 and 2, two distinguished candidates in mechanobiology, was found in the astrocytes of the optic nerve head (Choi et al., 2015). The presence of Caveolin-1 in astrocytes of the optic nerve and its increase under glaucomatous conditions *in vivo* lead to the assumption that the changes in biomechanical properties in the peripapillary sclera induced by an altered IOP are sensed by caveolae. Several studies could demonstrate the implication of Caveolin-1 in mechanosensing in various cell types. For instance, exposure of endothelial cells to chronic shear stress invokes the ERK pathway in a caveolae-dependent manner (Boyd et al., 2003; Park et al., 2000; Rizzo et al., 2003). Activated retinal astrocytes in the glaucomatous human retina in turn showed a prominent and persistent activation of ERK signaling (Tezel and Chauhan, 2003). In smooth-muscle-like cells, cyclic stretch can mediate the activation of Akt signaling, dependent on Caveolin-1 (Zhang et al., 2007). Caveolae provide a mechanism to extenuate an increase in membrane tension applied by acute mechanical stress by caveolae flattening (Sinha et al., 2011). Shear and cyclic stress increase the release of Nitric oxide (NO), a potent vasodilator and enhance endothelial nitric oxide synthase (eNOS) mRNA and protein (Harrison et al., 1996). eNOS hyperactivity is observed in Caveolin-1-deficient mice and caveolae are shown to be essential for shear stress induced eNOS activation in vascular endothelium (Drab et al., 2001; Yu et al., 2006; Zhao et al., 2002). In this study an increased substratum stiffness simulating biomechanical stress, led to an increase in Caveolin-1 expression. The substratum stiffness was selected ranging from 8 to 60 kPa, a range which was also described in an *in vivo* rabbit model, where the Young's modulus in the optic nerve head was increased from 14 kPa to 81kPa with the IOP increased from 15mmHg to 35mmHg (Zhang et al., 2024). Beside increased expression, an alteration in the localization of Caveolin-1 was also observed within the cells, similar to that reported in hepatic stellate cells (Lachowski et al., 2022). In other cell types, a mechanotransduction cascade has been established in which increased ECM stiffness induces Caveolin-1 expression, which in turn drives F-actin reorganization, YAP nuclear translocation, and subsequent CCN2/CTGF upregulation, ultimately promoting ECM remodeling (Moreno-Vicente et al., 2018). To further investigate a functional role of Caveolin-1 in astrocyte mechanotransduction, we performed loss-of-function experiments using Caveolin-1 siRNA under different substratum stiffness conditions. Following Caveolin-1 knockdown, collagen IV expression showed a consistent, albeit not statistically significant, increase at both 8 kPa and 50 kPa compared with corresponding controls, with a more pronounced effect on the stiffer substratum. In addition, pFAK was increased following Caveolin-1 knockdown on the 50 kPa substratum. While these findings should be interpreted with caution given the limited sample size, they suggest that reduced Caveolin-1 expression may enhance ECM- and focal adhesion-associated signaling responses to increased substratum stiffness, potentially through altered actin cytoskeletal organization and dynamics. Interestingly, downregulation of *Ccn2/Ctgf* in optic nerve astrocytes resulted in a reduction of Caveolin-1 mRNA whereas Caveolin-1 protein levels were increased, while Cavin-1 mRNA remained unchanged despite a reduction in Cavin-1 protein. Although the underlying mechanism remains to be determined, these findings may indicate altered caveolar organization or turnover following CCN2/CTGF depletion. Given that CCN2/CTGF exerts broad effects on ECM composition and cellular phenotype (Dillinger et al., 2022), the present data cannot distinguish whether this relationship reflects a direct regulatory link between CCN2/CTGF and caveolar components. Regardless of whether this relationship reflects a direct or indirect (ECM-mediated) mechanism, it is of particular interest in the context of POAG, given that CCN2/CTGF-driven ECM remodeling is itself a well-established feature of glaucomatous tissue remodeling in the optic nerve head (Dillinger et al., 2022). These findings therefore support a potential link between IOP-driven ECM/CCN2/CTGF-signaling and mechanosensory effects of Caveolin-1 in optic nerve astrocytes. Caveolae are highly dynamic membrane structures that respond to mechanical stress through rapid disassembly, resulting in the release of cavin proteins from the plasma membrane and their subsequent ubiquitination and proteasomal degradation, which could provide a potential explanation for the reduced Cavin-1 protein levels despite unchanged Cavin-1 transcript abundance (Tillu et al., 2015). At the same time, caveolar disassembly can increase the pool of non-caveolar Caveolin-1 at the plasma membrane, providing a potential explanation for the increase in Caveolin-1 protein despite reduced Caveolin-1 mRNA (Sinha et al., 2011). Given the established role of caveolae and Caveolin-1 in sensing and adapting to mechanical cues, and the close relationship between ECM composition, mechanotransduction and CCN2/CTGF signaling, these findings raise the possibility that CCN2/CTGF contributes to the regulation of caveolar homeostasis in optic nerve astrocytes. Moreover, Caveolin-1 and Cavin-1 are direct YAP/TAZ-TEAD target genes (Rausch et al., 2019), further linking caveolar dynamics to ECM-dependent mechanotransduction and suggesting a potential feedback mechanism between CCN2/CTGF, caveolae and YAP/TAZ signaling. Future studies should therefore investigate Caveolin-1/Cavin-1 localization and caveolae abundance, together with YAP/TAZ activity, to determine whether CCN2/CTGF depletion alters caveolae organization and mechanotransduction in optic nerve astrocytes. This places CCN2/CTGF not only as a downstream effector of Caveolin-1-mediated mechanosensing, but also as a positive regulator of its own upstream sensor. Whether this regulation occurs through direct transcriptional control, or indirectly via CCN2/CTGF-driven ECM remodeling that secondarily alters the mechanical microenvironment, cannot be resolved from the present data and warrants further investigation. Furthermore, Caveolin-1 was upregulated by CCN2/CTGF and TGF-β2. We have recently shown that there is a direct connection between biomechanical load and the expression of CCN2/CTGF in astrocytes of the glaucomatous glial lamina and lamina cribrosa (Dillinger et al., 2022). Extracellular matrix changes in the optic nerve head appear to be based on a disruption of the homeostatic balance of growth factors (Fuchshofer, 2011). One important growth factor responsible for fibrotic processes in the course of the disease is TGF-β2, which was shown to be elevated in the optic nerve head and the aqueous humor of patients suffering from POAG (Zode et al., 2011; Pena et al., 1999; Picht et al., 2001; Tripathi et al., 1994). Interestingly, the inhibition of mechanosensitive channels attenuated the TGF-β2 mediated GFAP expression and actin cytoskeleton remodeling in astrocytes of the optic nerve (Kirschner et al., 2021). The increased Caveolin-1 expression observed in the optic nerve head of βB1-CTGF1 mice may reflect an altered caveolar response associated with the glaucomatous environment. While astrocytes are a potential cellular source, Caveolin-1 is also highly expressed by vascular endothelial cells. However, given the relatively small proportion of endothelial cells within the optic nerve head, their contribution to the overall increase in Caveolin-1 is likely limited, although it cannot be completely excluded. Thus, the observed changes may reflect alterations in astrocyte biology together with potential vascular responses. Future cell-type-specific analyses will be required to determine the relative contribution of these cellular populations. In Caveolin-1 knockout mice, astrocytes showed a reduced capability to form a glial scar after an ischemic stroke which is consistent with the observation of interplay between ECM production and the presence of Caveolin-1. In the ischemic stroke model, wildtype littermates had a better recovery than Caveolin-1 knockout mice did, which led to the assumption that Caveolin-1 had a neuroprotective function in this case (Blochet et al., 2020). A role of Caveolin-1 to maintain the blood-brain barrier and to reduce neuroinflammation after a traumatic brain injury also suggests that Caveolin-1 is necessary to support neuronal survival after an injury (Niesman et al., 2014). Whether the potential beneficial role of Caveolin-1 extends to the optic nerve following glaucomatous injury and whether this may contribute to retinal ganglion cell survival and/or optic nerve degeneration remains to be determined. Future studies will be required to investigate these potential effects. These results suggest that pathological alterations in the optic nerve may be mediated by mechanotransduction pathways, potentially involving the mechanosensitive Caveolin-1 activated by an imbalance in growth factors such as TGF-β2 and CCN2/CTGF. Furthermore, a polymorphism in the Caveolin-1/Caveolin-2 gene was associated with both POAG and IOP (Thorleifsson et al., 2010; Wiggs et al., 2011; Chen et al., 2014; Huang et al., 2014; Hysi et al., 2014; Kim et al., 2015; Loomis et al., 2014; van Koolwijk et al., 2012). The implication in POAG pathology was furthermore proven by animal studies. It was shown that Caveolin-1 deficiency leads to IOP elevation and reduced aqueous humor drainage, accompanied by an increased sensitivity of outflow pathway cells to rupture from acute IOP elevation (Elliott et al., 2016; Kizhatil et al., 2016; Lei et al., 2016). However, to date, there is no information about glaucoma relevant changes in the optic nerve head associated with a Caveolin-1 deletion. It is tempting to speculate whether the increase of Caveolin-1 due to increasing mechanical load is harmful or beneficial to resident and surrounding tissues. Future studies must elucidate the direct role of Caveolin-1 in the pathogenesis of glaucoma in the optic nerve head. However, to our knowledge, we are the first to demonstrate a potential link between the mechanobiology of astrocytes in the glial lamina and Caveolin-1.

## 4 Material and methods

### 4.1 Animals

Transgenic βB1-CTGF1 mice were generated as described previously (Junglas et al., 2012) and compared to wildtype littermates. In brief, in this mouse line, the chicken βB1-crystallin promotor was used to direct high and specific expression of transgenes to lens fibers of the mouse eye. As the βB1-crystallin promotor is lens specific, the overexpression of CCN2/CTGF was only detected in the lens of the βB1-CTGF1 mouse line. *Ctgf*^*Coin*/*Coin*^ mice were generated as described previously by Canalis et al. (2010). *Ctgf*^*Coin*/*Coin*^ mice carry a conditional-by-inversion (COIN) allele in which an antisense lox66_SA-Egfp-polyA_lox71 element was inserted into the terminal region of *Ctgf* exon 2, maintaining normal CTGF expression until Cre-mediated irreversible inversion disrupts *Ctgf* expression (Canalis et al., 2010). Astrocytes isolated from *Ctgf*^*Coin*/*Coin*^ mice were subsequently treated with an adenoviral Cre system as described below. The experiments in this study were performed with mice of both sex. Mice were housed under standardized conditions of 63% air humidity and 21 °C room temperature. Feeding was *ad libitum*. Animals were kept at a 12h light/dark cycle (6a.m.−6p.m.). All procedures conformed to the tenets of the National Institutes of Health Guidelines on the Care and Use of Animals in Research, the EU Directive 2010/63/E and the ARVO Statement for the Use of Animals in Ophthalmic and Vision Research and were approved by the local authorities (54-2532.1-44/12; Regierung Oberpfalz, Bavaria, Germany).

### 4.2 Cell culture

Primary murine optic nerve astrocytes were isolated either from CD1 wildtype mice or *Ctgf*^Coin/Coin^ mice, characterized, and cultured according to our protocol which was previously published (Dillinger et al., 2022). Cells were cultured in DMEM/F-12 medium with 10% FCS and 1% penicillin/streptomycin until reaching confluency. For experiments, primary murine optic nerve astrocytes were used until passage 8. All experiments in this study were performed with at least three different cell lines. For the treatment with CCN2/CTGF or TGF-β2, astrocytes were seeded on 6-well plates and treated with 1 ng/ml TGF-β2, 50 ng/ml or 100 ng/ml CCN2/CTGF, or vehicle as controls for 24 h under serum-reduced (1% FCS) conditions. The concentration of CCN2/CTGF and TGF-β2 treatment was chosen in accordance with our previous findings (Quigley, 1987; Choi et al., 2015). For stiffness experiments, cells were seeded on 6-well plates with different stiffness (8 kPa, 50 kPa, glass) and grown for one week. 6-well with different stiffness were purchased from Matrigen (Irvine, USA) and pre-coated with collagen by the manufacturer. Processing of cells for the different analysis is depicted in the respective sections. Immortalized mural brain-derived endothelial 5 (b.End5) cells (Merck, 96091930) were cultured in high-glucose DMEM containing 4.5 g/L glucose, 2 mM glutamine, 1 mM sodium pyruvate, 10% fetal bovine serum (FBS), 1% penicillin/streptomycin, and 1% non-essential amino acids (NEAA) at 37 °C with 5% CO_2_.

### 4.3 siRNA transfection

For transfection, cells were incubated in serum reduced medium (1% FBS, 1% P/S) for 24h followed by incubation in transfection mix (200 μl DMEM/F12 (1% FBS, 1% P/S), 4 μl interferin, 0.5 μM Caveolin-1 siRNA (#s63423) or silencer select negative control No.1 (scrbl) siRNA (Invitrogen, Thermo Fisher Scientific, Waltham, USA)) for 24 h. Medium was then changed to DMEM/F12 (10% FBS, 1% P/S) and cells were harvested after 7 days.

### 4.4 Adenoviral gene transfer

Virus was generated as previously described (Junglas et al., 2012). For transduction, murine optic nerve astrocytes were isolated from CtgfCoin/Coin mice according to the protocol previously published (Dillinger et al., 2022) and were incubated with Ad5-Cre or Ad5-GFP control virus for 24 h with a titer of 2.3 x 10^−7^ focus-forming units per milliliter.

### 4.5 mRNA expression analysis

Total RNA was extracted form primary murine optic nerve astrocytes with TriFast™ (Peqlab, Erlangen, Germany) according to the manufacturer's instructions. In brief, 500μl TriFast™ were added to each well and cells were removed by using a cell scraper. Next, 200μl Chloroform were added, vortexed for 15s and centrifuged for 20 min at 12,00g at 4 °C to induce phase separation. The aqueous phase containing the total RNA was transferred into a new tube, precipitated with 100% isopropanol, washed twice with 75% ethanol, and dissolved in RNase-free water. Real-time RT-PCR was performed from total RNA using the qScript™ cDNA Synthesos Kit (Quanta Biosciences, Gaithersburg, United States) according to the manufacturer's instructions. Real-time RT-PCR was performed on a BioRad CFX Real-Time PCR Detection System (BioRad, München, Germany). Primers (Table 1) were designed to span exon-intron boundaries and purchased from Invitrogen.

**Table 1:** Oligonucleotide sequences for Real-time RT-PCR.

Oligonucleotide	Orientation	Species	Sequence 5‘to 3‘
*Caveolin1*	Reverse	Mus musculus	AACGACGACGTGGTCAAGA
*Caveolin1*	Forward	Mus musculus	CACAGTGAAGGTGGTGAAGG
*Caveolin2*	Reverse	Mus musculus	CCTCACCAGCTCAACTCTCA
*Caveolin2*	Forward	Mus musculus	CACATATTTGCTGATTTCAAAGAGA
*Caveolin3*	Reverse	Mus musculus	TGAGGACATTGTGAAGGTAGATTT
*Caveolin3*	Forward	Mus musculus	ACTTGGAGACGGTGAACGTG
*Cavin1*	Reverse	Mus musculus	CAGGGTCAACGTGAAGACC
*Cavin1*	Forward	Mus musculus	CCGGCAGCTTGACTTCAT
*Cavin2*	Reverse	Mus musculus	AGACTGGAGAACAACCACGC
*Cavin2*	Forward	Mus musculus	AGGGATCTCACTTTCTTCCTGG
*Cavin3*	Reverse	Mus musculus	GCTCAGGTGCAGCGGTTG
*Cavin3*	Forward	Mus musculus	GCTGGAATTTCAGTCTCCTCCTTG
*Cavin4*	Reverse	Mus musculus	GAGTGGTAATCTTCCAGGAGGACAT
*Cavin4*	Forward	Mus musculus	GGGCAGGCTTCTGTCTTTAAC
*Ctgf*	Reverse	Mus musculus	TGACCTGGAGGAAAACATTAAGA
*Ctgf*	Forward	Mus musculus	AGCCCTGTATGTCTTCACACTG
*Rpl32*	Reverse	Mus musculus	GCTGCCATCTGTTTTACGG
*Rpl32*	Forward	Mus musculus	TGACTGGTGCCTGATGAACT

### 4.6 Western blot analysis

Proteins were isolated following RNA isolation according to the manufacturer's instructions with TriFast™ (Peqlab, Erlangen, Germany). After removing the aqueous phase, containing the RNA, the remaining organic phase was used to isolate proteins. In brief, proteins were precipitated with isopropanol, washed three times with Guanidine hydrochloride, and once with 95% ethanol. Finally, proteins were dissolved in 1% SDS containing protease- and phosphatase inhibitor (Serva Electrophoresis GmbH, Heidelberg Germany). Protein concentration was determined by the bicinchoninic acid assay (Interchim, Montlucon Cedex, France). Proteins were separated by SDS-PAGE and transferred to either polyvinylidene difluoride (PVDF) or nitrocellulose membranes (Roche, Mannheim, Germany). Western Blot analysis was performed with specific antibodies as described previously (Quigley, 1987). Specific antibodies were used as follows: Caveolin-1 (rabbit, Cell Signaling, D46G3, 1:1000), Cavin1/PTRF (rabbit, ProteinTech, 18892-1-AP, 1:5000), Collagen IV (goat Life Technologie, AM4300, 1:1000), FAK (rabbit, Santa Cruz, sc-557, 1:1000), pFAK (rabbit, Santa Cruz, SC-11765-R, 1:1000), GAPDH (mouse, Life Technologie, AM4300, 1:1000), alpha-Tubulin (rabbit, Rockland, 600-401-880, 1:2000), Anti-mouse HRP (Jackson Immuno, 1:2000), Anti-goat HRP (donkey, abcam, 1:2000), Anti-rabbit HRP (goat, Cell Signaling, 1:2000).

### 4.7 Immunohistochemistry

Eyes of βB1-CTGF1 and wildtype mice at the age of 8 weeks were enucleated and fixed in 4% (w/v) paraformaldehyde (PFA) for 24h. The eyes were equilibrated in 10%, 20% and 30% sucrose, embedded in Tissue Tek optimal cooling temperature compound (SakuraFinetek Europe B.V., Zoeterwound, Netherland), and stored at −20 °C. Frozen 12 μm sections were cut on the cryostat. Cross sections of the retina and optic nerve, as well as tangential sections of the glial lamina were obtained. After blocking with 2% BSA, 0.2% cold water fish skin gelatin (CWFG) (Sigma Aldrich), 0.1% Triton-X-100 in 0.1M phosphate buffer, frozen sections were incubated with primary antibodies at 4 °C over-night (rabbit anti Caveolin-1, Cell Signaling, D46G3, 1:100; chicken anti GFAP, LSBio, LS-B4775, 1:1000). After subsequent washing with 0.1M Php, sections were incubated with secondary antibodies for 1h at RT (Donkey anti-rabbit Alexa FluorTM 647; Invitrogen, 1:1000; Goat anti-chicken Alexa FluorTM 488). Cell nuclei were counterstained via embedding with Mowiol mounting medium containing 1:10 DAPI.

### 4.8 Immunocytochemistry

Cells were washed twice with 1x PBS, fixed with 4% (w/v) PFA for 5 min, and washed again three times with PBS. For immunocytochemical staining cells were incubated for 1 h in 2% BSA, 0.2% CWFG, 0.1% Triton-X in 0.1 M phosphate buffer. Specific antibodies for staining were used as follows: Caveolin-1, Cavin-1, Vinculin+ secondary antibodies. Phalloidin-rhodamin (1:1,000, 1h at room temperature, Sigma-Aldrich, Taufkirchen, Germany) was used for labeling the actin cytoskeleton. As control for unspecific binding of secondary antibodies, negative controls were performed. Finally, 4,6-diamidino-2-phenylindole (Dapi, Vector Laboratories, Burlingame, CA, United States) diluted 1:10 in Mowiol mounting medium was added to counterstain nuclear DNA. The immunofluorescence was visualized using a Zeiss Axio Imager fluorescence microscope (Carl Zeiss AG, Jena, Germany).

### 4.9 Scanning electron microscopy

For scanning electron microscopy (SEM), 90,000 cells per well were seeded on glass coverslips in 6-well plates and cultured for 48 h under standard conditions. After 48 h, at approximately 80% confluence, cells were further cultured for 24 h in standard medium without FBS. Cells were washed three times with pre-warmed (37 °C) PBS and once with pre-warmed 0.1 M cacodylate buffer. Fixation was performed with electron microscopy fixative (2.5% paraformaldehyde, 2.5% glutaraldehyde in 0.1 M cacodylate buffer) for 4 h at 8 °C. Subsequently, cells were washed with 0.1 M cacodylate buffer, osmicated in 1% osmium tetroxide in distilled water for 30 min, and washed several times with distilled water. Dehydration was carried out through a graded ethanol series from 50% to 100%, followed by two 5 min incubations in a 1:1 mixture of 1,1,1,3,3,3-hexamethyldisilazane (HMDS) and 100% ethanol, and finally in 100% HMDS overnight. Dried samples were sputter-coated with platinum and examined using a field-emission scanning electron microscope (LEO 1530, Zeiss).

### 4.10 Quantification of immunofluorescent signal

Quantification of the %area of immunofluorescent labeling was performed with Fiji (ImageJ). To this end, a threshold was set from 30-255 for all images and subsequent measurements were conducted on the entire image. Measurements were conducted for 3 different cell lines per condition.

### 4.11 Statistical analysis

All experiments were repeated at least three times. Exact number of each experiment is indicated in the respective figure legend. Results are depicted as mean ± SD. Before the statistical analysis, the data were tested for normality, and the appropriate test was then selected. The corresponding statistical test is specified in the figure legend. Statistical analyses were performed using GraphPad Prism 9.5.1 (GraphPad Software, La Jolla, CA) with *p* ≤ 0.05 taken as statistically significant.

## Data Availability

The raw data supporting the conclusions of this article will be made available by the authors, without undue reservation.
