# Supplementary material for: Caveolin-1 defines astrocyte mechanobiology in the glial lamina of the optic nerve head
Source: Front Cell Neurosci. 2026 Oct 7;20:1909639. doi: 10.3389/fncel.2026.1909639 (PMC13683092; doi:10.3389/fncel.2026.1909639)
Supplement: Supplementary file 1 [file Table_1.docx]

**Original blots of the cropped images shown in the figures**

Figure 3B: Caveolin-1

Figure 3B: α-Tubulin

Figure 3D:Cavin-1

Figure 3D:Coomassie

Figure 4D: Caveolin-1

Figure 4D: α-Tubulin
