# Supplementary material for: Caveolin-1 defines astrocyte mechanobiology in the glial lamina of the optic nerve head
Source: Front Cell Neurosci. 2026 Oct 7;20:1909639. doi: 10.3389/fncel.2026.1909639 (PMC13683092; doi:10.3389/fncel.2026.1909639)
Supplement: Supplementary file 2 [file Table_2.docx]

**Suppl. Figure 1: relative mRNA expression of murine optic nerve head astrocytes *in vitro*.** mRNA levels were assessed by real-time RT-PCR experiments. (**A**) Rel. *Ccn2/Ctgf* mRNA levels in Ad5-GFP and Ad5-Cre treated astrocytes. (**B**) Rel. *Caveolin-1* mRNA levels in astrocytes following treatment with either scrbl siRNA or siRNA for *Caveolin-1*. *Rpl32* was used as the housekeeping gene. Unpaired t-test was used for statistical analysis. Data presented as mean ± SD. *P ≤ 0.05; **P < 0.01.

**Suppl. Figure 2: relative protein levels of collagen IV and pFAK/FAK in murine optic nerve head astrocytes cultured on increasing substratum stiffness following *Caveolin-1* silencing *in vitro*.** Protein levels were assessed by western blot experiments. (**A**) Rel. Collagen IV (Col IV) protein levels in astrocytes cultured on either 8 kPa or 50 kPa substratum and treated with either scrbl or *Caveolin-1* siRNA. (**B**) Rel. pFAK/FAK protein ratio in astrocytes cultured on either 8 kPa or 50 kPa substratum and treated with either scrbl or *Caveolin-1 (Cav1)* siRNA. GAPDH was used for protein normalization. Two-way ANOVA was used for statistical analysis. Data presented as mean ± SD. *P ≤ 0.05.
